# Comparison of trimodality therapy and neoadjuvant chemotherapy combined with radical cystectomy for the survival of muscle-invasive bladder cancer: a population-based analysis

**DOI:** 10.1186/s40001-023-01408-9

**Published:** 2023-10-11

**Authors:** Yi-Xin Zhou, Qian-Cheng Hu, Ya-Juan Zhu, Xiao-Li Mu, Ji-Yan Liu, Ye Chen

**Affiliations:** 1 Department of Biotherapy, Cancer Centre, West China Hospital, Sichuan University, 37 Guoxue Xiang Street, Chengdu, 610041 Sichuan China; 2 Gastric Cancer Center, Division of Medical Oncology, Cancer Center, Laboratory of Gastric Cancer, West China Hospital, Sichuan University, Chengdu, China; 3 Division of Abdominal Tumor Multimodality Treatment, Department of Radiation Oncology, Cancer Center, West China Hospital, Sichuan University, 37 Guoxue Xiang Street, Chengdu, 610041 Sichuan China

**Keywords:** Muscle-invasive bladder cancer, Trimodality therapy, Radical cystectomy, Neoadjuvant chemotherapy

## Abstract

**Background:**

Trimodality therapy (TMT) is a mature alternative to radical cystectomy (RC) for patients with muscle-invasive bladder cancer (MIBC) who seek to preserve their primary bladder or are inoperable due to comorbidities. To date, there has been increasing evidence of the effectiveness of TMT as an alternative to RC. In contrast, no literature has stated the effectiveness of neoadjuvant chemotherapy combined with RC (NAC + RC) compared with TMT.

**Objective:**

We aimed to compare the prognosis between patients receiving TMT and NAC + RC.

**Methods:**

The clinicopathological characteristics of patients with T2-4aN0M0 MIBC were obtained from the Surveillance, Epidemiology, and End Results (SEER) database. Univariate and multivariate Cox proportional hazards regression models and Kaplan‒Meier survival curves were used for the survival analysis. Propensity-score matching (PSM) was applied to determine the differences between the two groups. The primary outcome was cancer-specific survival (CSS), and the secondary outcome was overall survival (OS).

**Results:**

In total, 1,175 patients with MIBC who underwent TMT (n = 822) or NAC + RC (n = 353) were extracted from the Surveillance, Epidemiology, and End Results (SEER) database. After 1:1 PSM, the final patient sample included 303 pairs. The prognosis of patients receiving NAC + RC was significantly better than that of patients receiving TMT in both unmatched and matched cohorts (5-year CSS: before PSM, 75.4% vs. 50.6%, P < 0.0001; after PSM, 76.3% vs. 49.5%, P < 0.0001; 5-year OS: before PSM, 71.7% vs. 37.4%, P < 0.0001; after PSM, 71.7% vs. 31.4%, P < 0.0001). The survival advantages of NAC + RC remained remarkable in the stratified analysis of most factors after PSM. Multivariate Cox regression analysis showed that being older than 68 years old, unmarried, grade III/IV, T3-4a stage, and undergoing TMT independently correlated with poor OS.

**Conclusion:**

Thus, in this study, patients with MIBC receiving NAC + RC presented with a better prognosis than those receiving TMT.

## Introduction

Bladder cancer is the ninth most common cancer worldwide and the most common urinary tumor [1]. At the preliminary diagnosis, approximately one-third of patients are diagnosed with muscle-invasive bladder cancer (MIBC), and approximately 15% to 20% of nonmuscle-invasive bladder cancer (NMIBC) eventually progresses to MIBC [2]. Radical cystectomy (RC) has been considered the mainstay therapy for MIBC, with a reported 5-year overall survival (OS) of approximately 50% [3, 4]. Cisplatin-based neoadjuvant chemotherapy (NAC) has been applied in clinical practice to improve the survival benefits of RC [5]. However, about 59–70% of older patients with MIBC have age-related comorbidities such as renal impairment and cardiovascular or respiratory disease, which makes them unsuitable for surgery or chemotherapy [6] [7]. Considering the possible complications, about 1/3 of patients would choose bladder preservation rather than RC [8, 9].

For those patients with MIBC who rejected RC or NAC, trimodality therapy (TMT) has been investigated as an alternative, in which external beam radiotherapy (RT) and radiosensitizing chemotherapy are delivered after maximal transurethral bladder tumor resection (TURBT) [10]. Most patients receiving TMT could achieve a complete clinical response (cCR) of 70–80%, avoid salvage radical cystectomy, and provide long-term survival comparable to contemporary radical cystectomy series [11–14]. Although patients who experienced NAC + RC exhibited significant survival benefits compared to patients treated with RC only [5], the comparative effectiveness of TMT and NAC + RC remains unreported.

Therefore, based on the Surveillance, Epidemiology, and End Results (SEER) database, we aimed to compare the survival benefits of NAC + RC and TMT to provide an alternative treatment for clinicians and patients.

## Methods

### Patient cohort

**Fig. 1:**
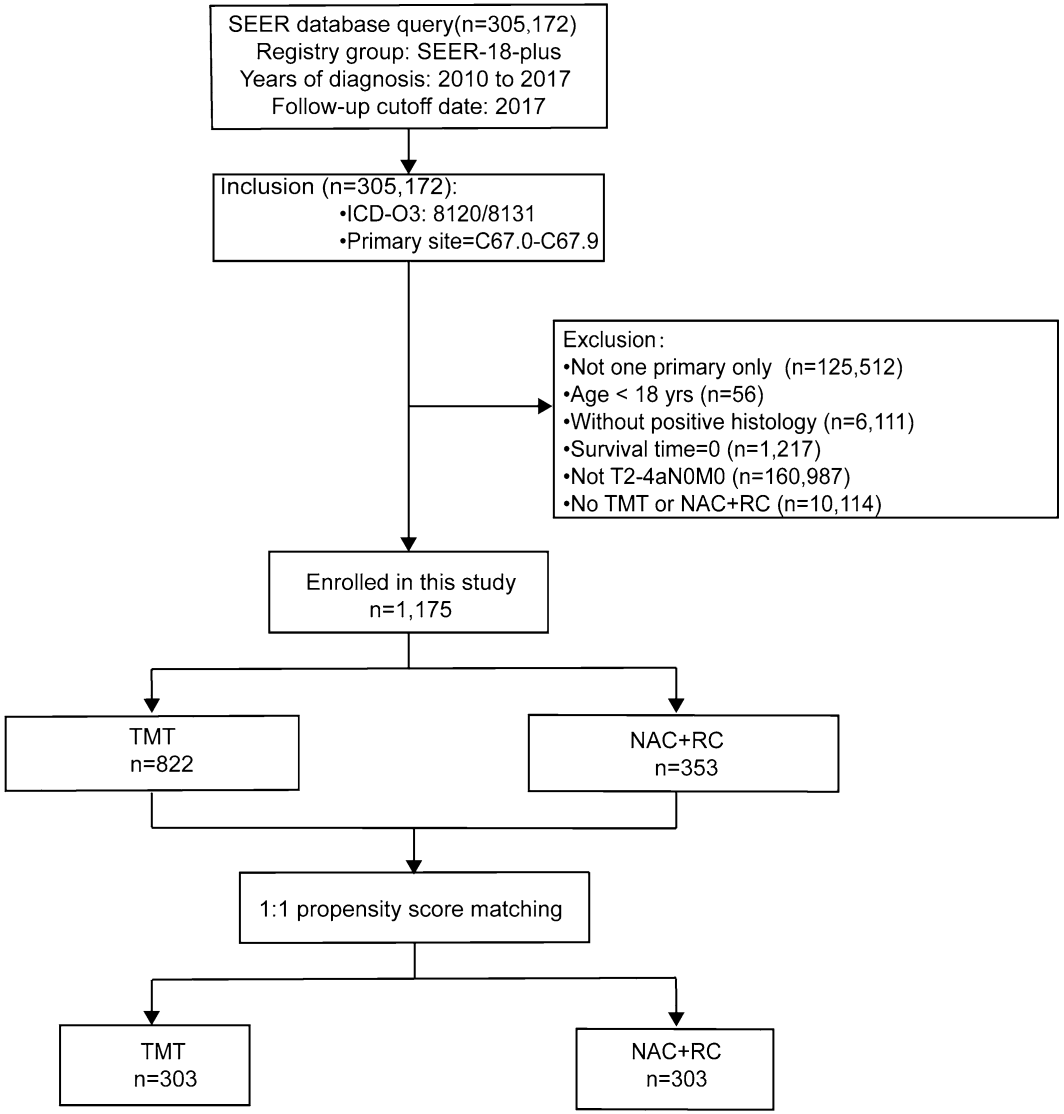
Flow diagram of selecting patients. TMT: trimodality therapy; NAC: neoadjuvant chemotherapy; RC: radical cystectomy

Data from 305,172 patients with muscle-invasive bladder cancer diagnosed from 1 January 2010 to 31 December 2017 were retrieved from the SEER-plus database. The International Classification of Diseases for Oncology, 3rd edition (ICD-O-3) morphology code was 8120/8131. Accordingly, 6 clinicopathological characteristics were extracted from the SEER program, including age, sex, race, marital status, and AJCC T stage (7th edition). Survival information regarding cancer-specific survival (CSS) and overall survival (OS) was also extracted. Patients who met the following criteria were excluded: (1) not one primary only; (2) age < 18 years; (3) without positive histology; (4) survival time = 0; (5) not T2–4aN0M0; and (6) no TMT or neoadjuvant chemotherapy (Fig. 1).

### Endpoint definition

The primary outcome was CSS, which was defined as the time from the date of diagnosis to the date of death from cancer. The secondary outcome was OS, which was defined as the time from the date of diagnosis to the date of death from any cause or the last follow-up.

### Statistical analysis

Patient data were extracted using SEER*Stat software version 8.3.9.0. Categorical variables are expressed as percentages. The chi-squared test was used to compare baseline characteristics between the two groups. To balance the confounding bias of the included cases, the meaningful clinicopathological prognostic factors of the multivariate analysis were included in the PSM. Nearest neighbor matching was performed at 1:1 in the TMT and NAC + RC groups. The Kaplan‒Meier method was used to generate cumulative survival curves, whereas the log-rank test was used for comparisons. Univariate and multivariate survival analyses were performed with the Cox proportional hazards model, and odds ratios (ORs) were computed with 95% confidence intervals (CIs). Statistical analysis in this study was performed using SPSS 25.0 and R (version 4.0.3). P < 0.05 was considered statistically significant.

## Results

### Baseline characteristics

A total of 1175 patients were enrolled in this study after exclusion, including 822 in the TMT group and 353 in the NAC + RC group (Fig. 1). After 1:1 PSM, the final patient sample included 303 pairs. The baseline characteristics of all enrolled patients are summarized in Table 1. In our research, the median age of all eligible patients was 68 years old. Most patients who underwent TMT were older than 68 years old, whereas more than 60% of patients who underwent NAC + RC were younger than 68 years old. Both therapies were more likely to occur in white males. Married patients were leaning toward receiving NAC + RC therapy. T stages were significantly different between the two groups, and patients with more advanced T stages were more likely to receive NAC + RC therapy (P < 0.001). Most variables were comparable between the two groups after PSM.

**Table 1:** The demographic and clinical characteristics of eligible TMT and NAC + RC patients before and after the propensity score match

Variables	Data before PSM			Data after PSM		
	TMT N = 822	NAC + RC N = 353	P	TMT N = 303	NAC + RC N = 303	P
Age						
< 68 years	198 (24.1)	217 (61.5)	< 0.001	157 (51.8)	167 (55.1)	0.464
≥ 68 years	624 (75.9)	136 (38.5)		146 (48.2)	136 (44.9)	
Sex						
Male	619 (75.3)	261 (73.9)	0.673	233 (76.9)	221 (72.9)	0.303
Female	203 (24.7)	92 (26.1)		70 (23.1)	82 (27.1)	
Race						
White	712 (86.6)	315 (89.2)	0.104	261 (86.1)	271 (89.4)	0.117
Black	65 (7.9)	16 (4.5)		24 (7.9)	12 (4.0)	
Other/Unknown	45 (5.5)	22 (6.2)		18 (5.9)	20 (6.6)	
Marital status						
Married	459 (55.8)	224 (63.5)	0.018	182 (60.1)	192 (63.4)	0.452
Unmarried/unknown	363 (44.2)	129 (36.5)		121 (39.9)	111 (36.6)	
Grade						
I/II	19 (2.3)	7 (2.0)	0.939	4 (1.3)	7 (2.3)	0.659
III/IV	742 (90.3)	320 (90.7)		278 (91.7)	275 (90.8)	
Unknown	61 (7.4)	26 (7.4)		21 (6.9)	21 (6.9)	
T stage						
T2	726 (88.3)	232 (65.7)	< 0.001	222 (73.3)	232 (76.6)	0.257
T3–4a	62 (7.5)	93 (26.3)		56 (18.4)	56 (18.4)	
Unknown	34 (4.1)	28 (7.9)		25 (8.3)	15 (5.0)	

TMT: trimodality therapy; NAC: neoadjuvant chemotherapy; RC: radical cystectomy

### Survival analysis

**Fig. 2:**
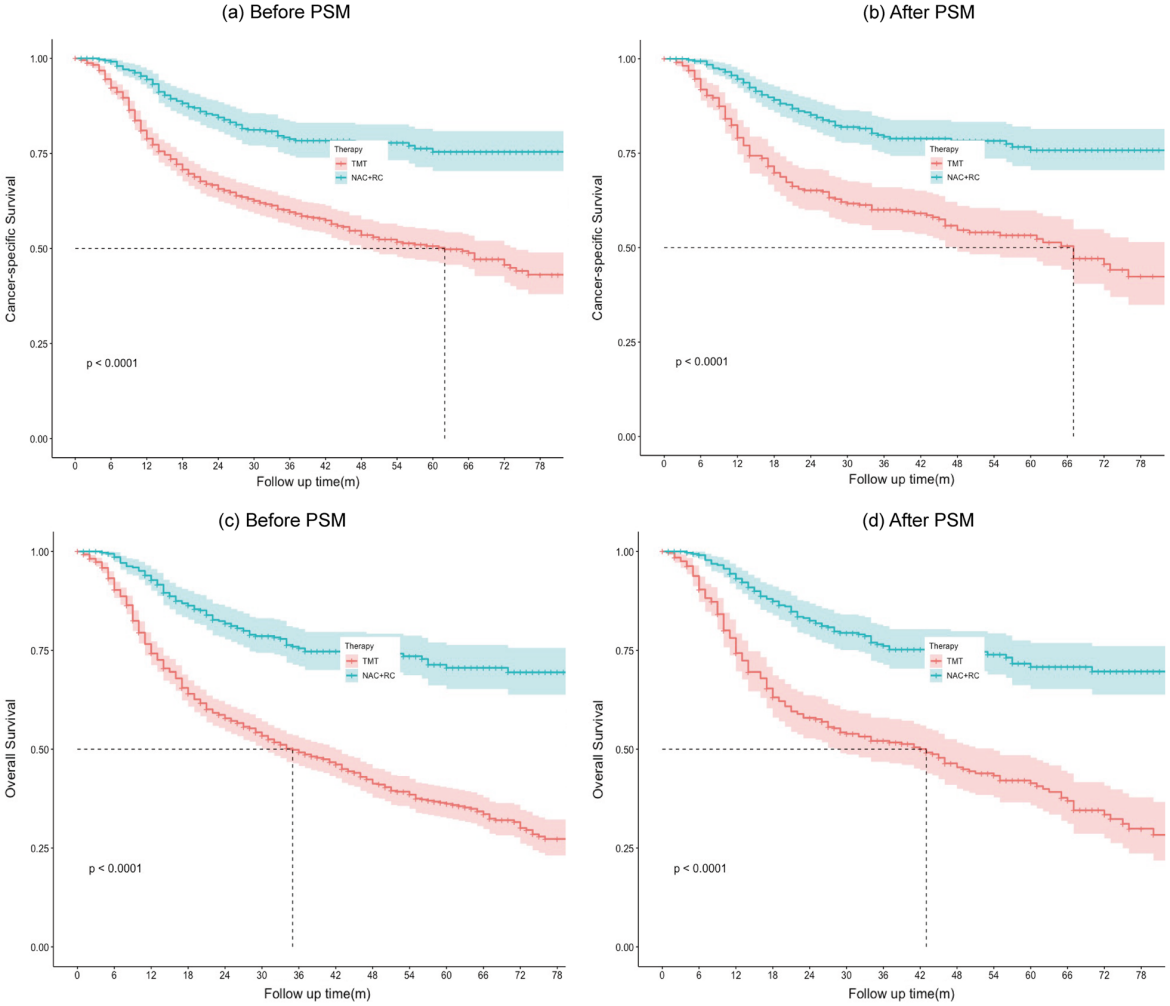
Survival curves prior to the match and matched cohorts of cancer-specific survival (**a**, **c**) and overall survival (**b**, **d**). TMT: trimodality therapy; NAC: neoadjuvant chemotherapy; RC: radical cystectomy

Survival curves are presented in Fig. 2. The median follow-up was 30 months before PSM. Regarding CSS before PSM, patients who received NAC + RC had a better 5-year survival than those who received TMT (75.4% vs. 50.6%, P < 0.0001). Regarding 5-year OS before PSM, a similar trend was observed (71.7% vs. 37.4%, P < 0.0001). The median follow-up was 34 months in the propensity-score-matched cohort. After PSM, patients who underwent TMT still had shorter 5-year CSS (49.5% vs. 76.3%, P < 0.0001) and 5-year OS (31.4% vs. 71.7%, P < 0.0001) than those who underwent NAC + RC.

**Fig. 3:**
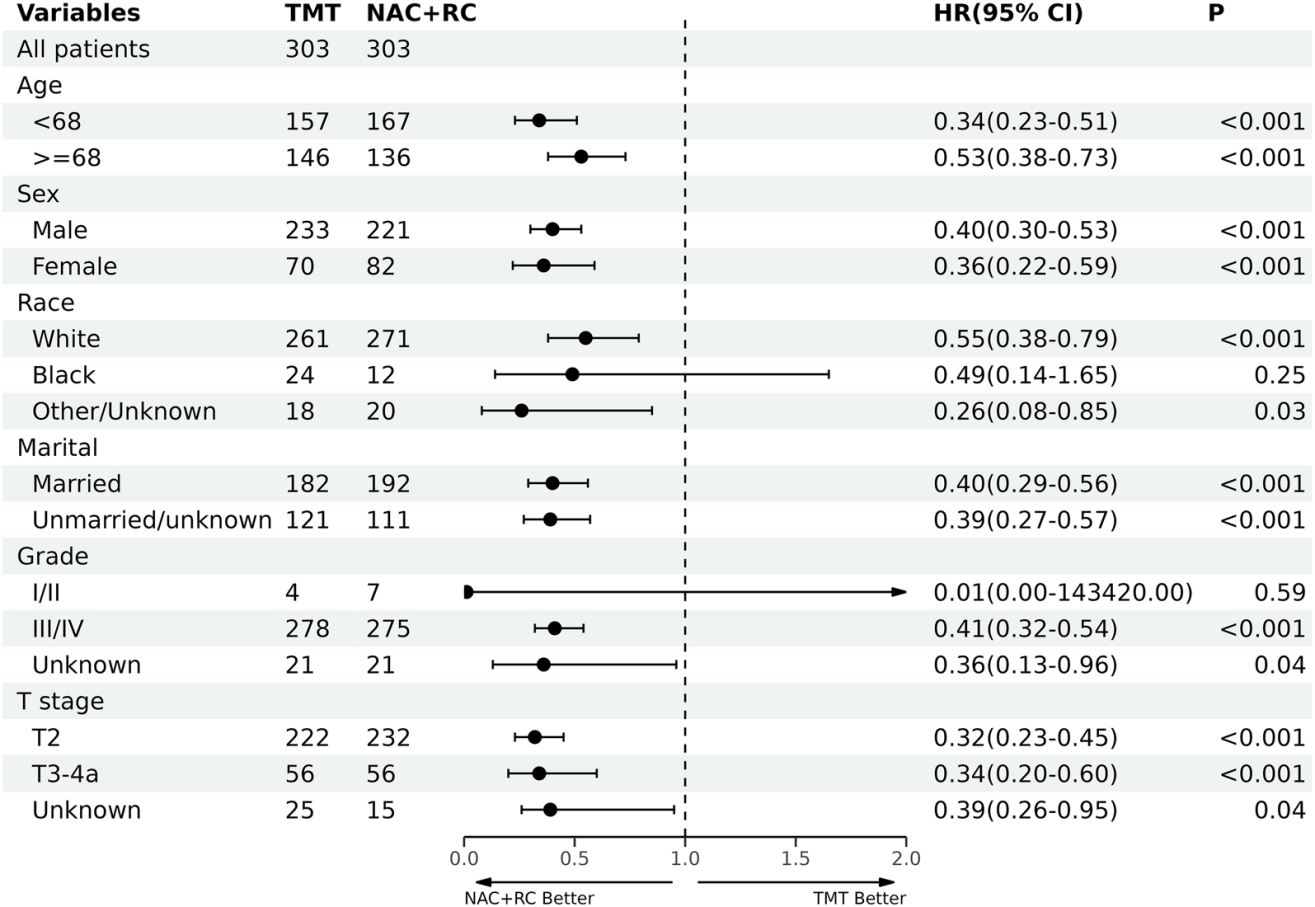
Stratified analyses of the 1:1 matched cohort (overall survival). TMT: trimodality therapy; NAC: neoadjuvant chemotherapy; RC: radical cystectomy; CI: confidence interval; HR: hazard ratio

Stratified analysis was performed to identify further the subgroups' different survival patterns of patients who underwent TMT and NAC + RC. Among most subgroups, patients who underwent NAC + RC showed a significantly better CSS than patients who received TMT in most stratified factors (Fig. 3). No significant difference was identified among specific subgroups, such as black race and grade I/II. However, it is important to note that this lack of significance may be attributed to the small sample size within these subgroups.

### Univariate and multivariate Cox analysis of OS

To determine the impact of various elements on survival, univariate and multivariate analyses of the OS of all patients before and after PSM were performed separately (Tables 2, 3). In the univariate analysis, before PSM, patients who were unmarried or older than 68 years were more likely to have a poor prognosis. No sexual preference was discovered in the survival analysis. Patients with grade III/IV and stage T3–4a tended to have worse prognoses, as did patients receiving TMT instead of NAC + RC. In the multivariate analysis of all patients, age older than 68, unmarried or unknown marital status, stage T3–4a, and receiving TMT remained significantly correlated with poor prognosis. After PSM, a similar pattern was identified, and these factors above were still identified as independent poor prognostic factors.

**Table 2:** Univariate and multivariate Cox analysis of overall survival before PSM

Characteristics	Univariable			Multivariable		
	Hazard ratio	95% CI	P	Hazard ratio	95% CI	P
Age						
< 68 years	Reference			Reference		
≥ 68 years	2.046	1.692–2.473	< 0.001	1.610	1.320–1.963	< 0.001
Sex						
Male	Reference			Reference		
Female	1.508	0.878–1.276	0.557	0.976	0.804–1.186	0.809
Race						
White	Reference			Reference		
Black	1.364	1.020–1.825	0.036	1.267	0.943–1.701	0.116
Other/Unknown	1.002	0.697–1.441	0.991	0.986	0.686–1.419	0.941
Marital status						
Married	Reference			Reference		
Unmarried/unknown	1.384	1.175–1.631	< 0.001	1.326	1.118–1.572	< 0.001
Grade						
I/II	Reference			Reference		
III/IV	1.949	1.008–3.768	0.047	2.099	1.084–4.064	0.028
Unknown	1.962	0.955–4.033	0.067	2.159	1.048–4.444	0.037
T stage						
T2	Reference			Reference		
T3–4a	1.044	0.846–1.288	0.043	1.488	1.194–1.854	< 0.001
Therapy						
TMT	Reference			Reference		
NAC + RC	0.334	0.267–0.418	< 0.001	0.358	0.281–0.457	< 0.001

TMT: trimodality therapy; NAC: neoadjuvant chemotherapy; RC: radical cystectomy; CI: confidence interval; HR: hazard ratio

**Table 3:** Univariate and multivariate Cox analysis of OS after PSM

Characteristics	Univariable			Multivariable		
	Hazard ratio	95% CI	P	Hazard ratio	95% CI	P
Age						
< 68 years	Reference			Reference		
≥ 68 years	1.974	1.538–2.535	< 0.001	1.929	1.482–2.510	< 0.001
Sex						
Male	Reference			Reference		
Female	1.103	0.832–1.462	0.496	1.136	0.845–1.527	0.309
Race						
White	Reference			Reference		
Black	1.367	0.873–2.139	0.172	1.079	0.682–1.702	0.745
Other/Unknown	0.981	0.572–1.685	0.946	0.978	0.567–1.685	0.935
Marital status						
Married	Reference			Reference		
Unmarried/unknown	1.529	1.196–1.956	< 0.001	1.326	1.118–1.572	< 0.001
Grade						
I/II	Reference			Reference		
III/IV	7.054	0.989–50.307	0.047	6.444	0902–44.023	0.063
Unknown	7.596	1.013–56.940	0.049	7.114	0.947–53.414	0.056
T stage						
T2	Reference			Reference		
T3–4a	1.286	0.944–1.751	0.003	1.068	0.775–14,731.854	< 0.001
Therapy						
TMT	Reference			Reference		
NAC + RC	0.311	0.237–0.408	< 0.001	0.350	0.270–0.453	< 0.001

TMT: trimodality therapy; NAC: neoadjuvant chemotherapy; RC: radical cystectomy; CI: confidence interval; HR: hazard ratio

## Discussion

RC has shown great survival effectiveness for MIBC patients but may not be eligible for all patients due to some patients having reached quite advanced stages or preferring to retain the bladder [15, 16]. In recent decades, TMT has been increasingly considered as an alternative to RC [17, 18]. However, the clinical benefits of TMT compared with NAC + RC are not yet clear. To provide solid evidence to guide clinicians and patients in choosing therapies, we compared the survival benefits of MIBC patients who were treated by NAC + RC and TMT.

In this research, we found that TMT was more commonly applied in elderly patients, whereas NAC + RC was preferred in younger patients (age ≥ 68: 75.9% vs. 38.5%, P < 0.001). Patients with more advanced T stages were more likely to receive NAC + RC rather than TMT (T3–4a: 26.3% vs. 7.5%, P < 0.001). This may be caused by selection bias in clinical practice. It is not difficult to understand that patients with older age may have poorer surgical tolerance, and patients with more advanced T stage need to receive a more thorough surgical procedure. Because patients with MIBC exhibited heterogeneity between the NAC + RC and TMT groups, we performed propensity matching to reduce selection bias. After PSM, all characteristics were balanced between the two groups, including age (age ≥ 68: 51.8% vs. 55.1%, P = 0.464), T stages (T3–4a: 18.4% vs. 18.4%, P = 0.257), etcetera. We also identified elder age, unmarried, advanced T stages, and received TMT instead of NAC + RC were independent poor prognostic factors for MIBC patients. Age has been reported to be correlated with more adverse outcomes and poor prognosis among patients received RC [19]. Previous studies have reported that T stage is the second most vital predictor of MIBC survival outcome after RC [20–22]. Based on the high risk of mortality in patients with advanced T stages, neoadjuvant therapy should be considered.

Our results indicated that both the 5-year CSS and OS were better in patients who underwent NAC + RC than in those who underwent TMT. Then, we performed a subgroup analysis to determine the group of patients best suited for each type of local treatment. The analysis further revealed that among most patients, NAC + RC was correlated with a better prognosis, except for black patients and patients with grade III/IV, which showed no significant difference between TMT and NAC + RC. These results suggested that NAC + RC is still a more effective treatment for most patients.

In most patients with high surgical risk or who are unwilling to resect the bladder, treatments that could preserve the bladder are recognized as optional treatments for RC [23, 24]. It is now believed that among these treatments for bladder preservation, TMT not only exhibits the best oncological effect but can also be selected to improve QOL by retaining bladder function in the elderly population [11, 25, 26]. Another retrospective study has previously reported that the elder patients were more likely to take TMT instead of RC (percentages of patients with age ≥ 80: 45.9% vs. 24.7%) [27], which is consistent with our study. The choice of patients with TMT has gradually increased, and most of them were elderly people with possibly more comorbidities, which may be the underlying reason for the low survival rates of TMT in our research. However, as our results indicated, NAC + RC still has a better prognosis even after population propensity matching (OS: HR, 0.33; 95% CI 0.27–0.48). Previous research based on SEER database of 3200 older adults (aged ≥ 66 years) with clinical stage T2 to T4a bladder cancer also stated that compared with RC, patients who underwent TMT had significantly decreased OS and CSS (OS: HR, 1.49, 95% CI 1.31–1.69), and the median total costs were substantially higher for TMT than for RC [27]. Another retrospective research of MIBC patients also suggested that TMT was associated with a significant adverse impact on long-term OS (HR 1.37, 95% CI 1.16–1.59) [28]. In addition, the effectiveness of TMT requires not only professional oncologists and radiotherapists but also specialists in bladder surgeries who can successfully perform a TURBT. Therefore, for those patients who meet the requirements of TMT, there should be an opportunity to discuss all possible treatment regimens.

Although NAC + RC has shown considerable efficacy in our study, around 50% of patients cannot receive cisplatin chemotherapy due to various reasons such as other health conditions, impaired kidney function, or previous contraindications [29, 30]. Current studies also indicated the possible vital role of neoadjuvant or adjuvant immunotherapy including PD-1, PDL-1, and CTLA-4 inhibitors has shown efficacy in treating MIBC [30, 31]. So far, many clinical trials discovering the efficacy of adjuvant or neoadjuvant immunotherapy alone or in combination with chemotherapy have shown promising results with acceptable safety profiles in MIBC [32–36]. As reported, neoadjuvant immunotherapy achieved a higher pathological complete response rate than neoadjuvant chemotherapy (42–46% vs 20–40%) [32, 33]. The increasing use of immunotherapy in the neoadjuvant treatment of MIBC indicates its potential role in TMT, either as a standalone treatment or in combination with chemotherapy. Relevant clinical studies have been carried out and the results are worth expected (NCT05072600, NCT05531123).

However, the study contains limitations. First, the lack of external verification by other populations may reduce the universality of our conclusion. Second, our study is retrospective, and excluding some patients with MIBC due to missing data could introduce bias, although we tried to control potential bias by using propensity score matching. Third, SEER doesn't have disease-free survival data, so we chose CSS and OS as alternatives. Finally, the SEER database does not contain specific information on the doses, techniques, or sites of radiotherapy, either the exact chemotherapy drugs.

## Conclusion

In general, compared with patients receiving TMT, patients receiving NAC + RC had a markedly better prognosis. Older patients and patients with earlier T stages were more likely to take TMT than NAC + RC. Being older than 68 years old, being unmarried, grade III/IV, T3–4a stage, and undergoing TMT were identified as independent poor prognostic factors in all MIBC patients. These findings are primary and underline the requirement for randomized controlled trials to compare TMT with NAC + RC.

## Data Availability

The datasets generated or analysed during this study are available in the SEER database.
